# Proteasome Inhibition Activates Autophagy-Lysosome Pathway Associated With TFEB Dephosphorylation and Nuclear Translocation

**DOI:** 10.3389/fcell.2019.00170

**Published:** 2019-08-22

**Authors:** Chunyan Li, Xin Wang, Xuezhi Li, Kaixin Qiu, Fengjuan Jiao, Yidan Liu, Qingxia Kong, Yan Liu, Yili Wu

**Affiliations:** ^1^Cheeloo College of Medicine, Shandong University, Jinan, China; ^2^Shandong Collaborative Innovation Center for Diagnosis, Treatment and Behavioral Interventions of Mental Disorders, Institute of Mental Health, Jining Medical University, Jining, China; ^3^Shandong Key Laboratory of Behavioral Medicine, School of Mental Health, Jining Medical University, Jining, China; ^4^Department of Neurology, Affiliated Hospital of Jining Medical University, Jining, China

**Keywords:** ubiquitin-proteasome pathway, autophagy-lysosome pathway, TFEB, TFEB nuclear translocation, TFEB dephosphorylation

## Abstract

Ubiquitin-proteasome pathway (UPS) and autophagy-lysosome pathway (ALP) are the two major protein degradation pathways, which are critical for proteostasis. Growing evidence indicates that proteasome inhibition-induced ALP activation is an adaptive response. Transcription Factor EB (TFEB) is a master regulator of ALP. However, the characteristics of TFEB and its role in proteasome inhibition-induced ALP activation are not fully investigated. Here we reported that the half-life of TFEB is around 13.5 h in neuronal-like cells, and TFEB is degraded through proteasome pathway in both neuronal-like and non-neuronal cells. Moreover, proteasome impairment not only promotes TFEB accumulation but also facilitates its dephosphorylation and nuclear translocation. In addition, proteasome inhibition-induced TFEB accumulation, dephosphorylation and nuclear translocation significantly increases the expression of a number of TFEB downstream genes involved in ALP activation, including microtubule-associated protein 1B light chain-3 (LC3), particularly LC3-II, cathepsin D and lysosomal-associated membrane protein 1 (LAMP1). Furthermore, we demonstrated that proteasome inhibition increases autophagosome biogenesis but not impairs autophagic flux. Our study advances the understanding of features of TFEB and indicates that TFEB might be a key mediator of proteasome impairment-induced ALP activation.

## Introduction

Ubiquitin-proteasome pathway (UPS) and autophagy-lysosome pathway (ALP), the two major pathways for protein degradation, are crucial for proteostasis (Kaganovich et al., 2008). They play a critical role in the maintenance of physiological functions, e.g., cell cycle, apoptosis, stress response, etc. Increasing evidence indicates that there is cross-talk between UPS and ALP. Moreover, the dysregulation of UPS and ALP is observed in a number of diseases, which plays a pivotal role in the pathogenesis of diseases, e.g., Alzheimer’s disease. Growing evidence indicates that proteasome inhibition-induced ALP activation is an adaptive response (Albornoz et al., 2019). However, the mechanism remains elusive. UPS is the principal system for degradation of unneeded or misfolded proteins in mammalian cells (Etlinger and Goldberg, 1977; Ciehanover et al., 1978). The ubiquitination of target proteins is sequentially catalyzed by ATP-dependent ubiquitin-activating enzyme E1, ubiquitin-conjugating enzyme E2, and ubiquitin-protein ligase E3, gaining the access to proteasomes (Haas et al., 1982). Proteasome consists of a 20S central catalytic complex and two 19S regulatory complexes (Hough et al., 1987; Arrigo et al., 1988; Dong et al., 2019). The 19S complexes deubiquinate and unfold the target proteins, allowing the proteins entering into the 20S proteasome, where they are rapidly degraded by various proteases (Hough et al., 1987; Arrigo et al., 1988; Dong et al., 2019). Proteasomes exist in both nucleus and cytoplasm, contributing to the degradation of cytosplasmic and nuclear proteins (Peters et al., 1994). UPS impairment is observed in a number of neurodegenerative diseases, including Alzheimer’s disease, Parkinson’s disease, Huntington’s disease, etc., which promotes the pathogenesis of these diseases. First, UPS impairment leads to the dysregulation of gene expression by reducing the degradation of transcriptional factors, e.g., NF-κB, HIF-1α, NFAT, etc., which contributes to the dysregulation of neurodegenerative disease-associated proteins, e.g., BACE1, RCAN1, TMP21, etc. (Sun et al., 2006; Liu et al., 2011; Ly et al., 2013; Wu and Song, 2013; Wu et al., 2015). In addition, various neurodegenerative disease-associated proteins are degraded via UPS, e.g., BACE1, RCAN1, TMP21, etc. (Qing et al., 2004; Liu et al., 2008; Liu et al., 2009; Wang et al., 2012). Moreover, reduced proteasome activity exacerbates the accumulation of damaged or misfolded proteins, e.g., tau and α-synuclein, which might further contribute to the pathogenesis of neurodegenerative diseases (David et al., 2002; Poppek et al., 2006; Alvarez-Castelao et al., 2014).

Autophagy-lysosome pathway is mainly responsible for the degradation of aged or damaged organelles and long-lived proteins, contributing to intracellular quality control. Autophagy is a process delivering intracellular constituents to lysosomes for degradation, which is characterized by the formation of autophagosome (Suzuki and Ohsumi, 2007). Autophagy is categorized into three types, microautophagy, chaperone-mediated autophagy (CMA) and macroautophagy, with distinct mechanistic features. Macroautophagy pathway is the major type of autophagy, which has been greatly characterized compared with the other two types. Macroautophagy (autophagy hereafter) starts with a cup-shaped isolated membrane, which engulfs substrates, protein aggregates or organelles to form a spherical double-membraned structure called autophagosome (Parzych and Klionsky, 2014). A number of autophagy-related proteins are implicated in the formation of autophagosome, e.g., microtubule-associated protein 1B light chain-3 (LC3). Mature LC3 is called as LC3-I, while phosphatidylethanolamine-conjugated LC3-I is called as LC3-II. As LC3-II is persistently associated with autophagosomes, increased LC3-II is commonly used as a marker of autophagy activation (Ohsumi, 2014). Lysosome, formed of a membrane-enclosed vacuole, is an acidic cell apparatus. Lysosomal-associated membrane protein 1 (LAMP1) and lysosomal-associated membrane protein 2 (LAMP2) account for approximately 50% of all lysosome membrane proteins, which act as a marker of the amount and integrity of lysosome. Lysosome mainly contains various acidic proteases, e.g., cathepsin D, which contribute to the degradation of proteins or organelles. Autophagosomes fuse with lysosomes to form autophagolysosomes, in which the cargo substrates, protein aggregates or organelles are degraded by acidic proteases in the lysosome. ALP is constitutively maintained at the basal level. It is regulated by cellular stimuli (e.g., nutrient fluctuation, starvation, and hypoxia), damaged organelles, misfolded protein accumulation-induced unfolded protein response, pathogens, etc. For example, nutrient depletion inactivates mechanistic target of rapamycin (mTOR), contributing to ALP activation (Meley et al., 2006; Wullschleger et al., 2006; Hardie, 2007; Napolitano et al., 2018). Dysregulation of ALP is implicated in the pathogenesis of diseases, including Alzheimer’s disease, Parkinson’s disease, Huntington’s disease, etc. For example, various Alzheimer’s disease-associated molecules are degraded by ALP, such as APP and BACE2 (Liu et al., 2013; Yang et al., 2013).

Autophagy-lysosome pathway is regulated at transcriptional level by a number of transcriptional factors. For example, transcription factor EB (TFEB), transcription factor binding to immunoglobulin heavy constant mu enhancer 3 (TFE3), forkhead box O (FOXO) and E2 transcription factor (E2F) contribute to the regulation of genes implicated in autophagosome induction, formation and maturation, lysosome biogenesis, lysosome function, etc. TFEB, a member of the basic helix-loop-helix leucine-zipper family of transcription factors, is a master regulator of ALP by controlling the expression of genes required for autophagosome formation, lysosome biogenesis and lysosome function (Settembre et al., 2011). The activity of TFEB mainly depends on its phosphorylation status and cytoplasm-nucleus shuttling (Puertollano et al., 2018). The phosphorylated TFEB is mainly located in the cytoplasm under physiological conditions, which is the inactive form of TFEB. Under stress conditions, TFEB is activated by kinase inactivation- and phosphatase activation-mediated dephosphorylation and translocates to the nucleus. For example, upon starvation or lysosomal stress, inactivation of mTOR and concomitant activation of the phosphatase calcineurin promotes TFEB dephosphorylation, resulting in its nuclear translocation (Medina et al., 2015; Napolitano et al., 2018). Subsequently, nuclear TFEB binds to the coordinated lysosomal expression and regulation (CLEAR) element, upregulating the expression of its target genes, e.g., LC3, cathepsin D, and LAMP1, contributing to the increasing activity of ALP (Mizushima, 2007; Zheng et al., 2009; Behrends et al., 2010). A recent study showed that TFEB acetylation also contributes to the activation of lysosome activity (Zhang et al., 2018). In addition, Sha et al. reported that TFEB is degraded by proteasome pathway with a half-life of 4–6 h in Hela cells (Sha et al., 2017). The feature of TFEB in neuronal-like cells has not been characterized although both TFEB and TFEB-mediated ALP play a pivotal role in the maintenance of central nervous system (CNS) functions. Moreover, the dysregulation of TFEB and TFEB-mediated ALP contributes the pathogenesis of various neurodegenerative diseases including Alzheimer’s disease and Parkinson’s disease, while increasing TFEB expression or activation attenuates the pathogenesis of neurodegenerative diseases (Martini-Stoica et al., 2016; Cortes and La Spada, 2019).

Increasing evidences indicate that there is crosstalk between UPS and ALP (Korolchuk et al., 2009a, b; Nam et al., 2017). Although proteasome inhibition-induced ALP activation is well documented, underlying mechanisms remain elusive. In this study, we reported that the half-life of TFEB is around 13.5 h in neuronal-like cells, and TFEB is degraded through proteasome pathway in both neuronal-like and non-neuronal cells. Moreover, proteasome impairment not only promotes TFEB accumulation but also facilitates its dephosphorylation and nuclear translocation. In addition, proteasome inhibition-induced TFEB accumulation, dephosphorylation and nuclear translocation significantly increases the expression of a number of TFEB downstream genes involved in ALP activation, including LC3, particularly LC3-II, cathepsin D, and LAMP1. Furthermore, we demonstrated that proteasome inhibition increases autophagosome biogenesis but not impairs autophagic flux. Our study advances the understanding of features of TFEB and indicates that TFEB might be a key mediator of proteasome impairment-induced ALP activation.

## Materials and Methods

### Cell Culture

Human embryonic kidney cells HEK293 and human neuroblastoma cells SH-SY5Y were cultured in high-glucose Dulbecco’s modified Eagle’s medium supplemented with 10% fetal bovine serum and 100 U/mL penicillin-streptomycin. All cells were maintained in an incubator at 37°C with 5% CO_2_.

### Pharmacological Treatment

Protein half-life was determined by using a 100 μg/mL cycloheximide (CHX) for 0, 4, 8, 12, 16 or 24 h. Proteasome inhibitor MG-132 was applied for 24 h with the concentration of 0, 10, 15 or 20 μM. In time-course experiments, 15 μM MG-132 was applied for 0, 3, 6, 12 or 24 h. 300 nM Bafilomycin A1 (Baf-A1) was applied for 4 h. Baf-A1 was purchased from Abcam and other drugs were purchased from Sigma-Aldrich. The details were described in previous studies (Settembre et al., 2011; Liu et al., 2013; Wu and Song, 2013; Medina et al., 2015).

### Western Blot Analysis

Cells were washed with PBS and lysed in RIPA-Doc buffer (Tris–HCl, 50 mM; NaCl, 150 mM; Triton X-100, 1%; deoxycholate, 1%; and SDS, 0.1%; supplemented with protease inhibitors and phosphatase inhibitors). Cell lysates were centrifuged at 12000 rpm for 30 min to pellet the cellular debris. The cytoplasmic proteins and the nuclear proteins were extracted according to instructions of nuclear and cytoplasmic extraction reagents kit (Beyotime, Beijing, China). The supernatant was diluted in 5x SDS-sample buffer and boiled. After resolved in 10% tris–glycine SDS-PAGE, the proteins were transferred to polyvinylidene fluoride (PVDF-FL) membranes. The membranes were blocked with 5% non-fat milk dissolved in 0.05% Tris–buffered saline with Tween 20 (TBST) for 1 h at room temperature, and incubated with primary antibodies overnight at 4°C. After incubation, the membranes were washed with TBST and incubated with secondary antibodies, anti-rabbit antibody or anti-mouse antibody, for 1 h at room temperature. The quantification was performed by using ImageJ. The following antibodies were used: anti-TFEB (cat. 4240, 1:1000) from Cell Signaling Technologies; anti-β-actin (TA-09, 1:5000) from Zhongshan Golden Bridge Biotechnology; anti-LAMP-1 (H4A3, cat. sc-20011, 1:500) and anti-TMP21 (A-7, cat. sc-137003, 1:500) from Santa Cruz Biotechnology; anti-LC3 (cat. NB100-2220, 1:2000) from Novus Biologicals; anti-β-tubulin (cat. WL01931, 1:500) from Wanleibio; anti-Lamin B (cat. WL01775, 1:500) from Wanleibio; anti-rabbit IgG (2B-2301, 1:5000) or anti-mouse IgG (ZB-2305, 1:5000) from Zhongshan Golden Bridge Biotechnology.

### Confocal Microscopy

HEK293 cells transiently transfected with TFEB-GFP were seeded on coverslips. 24 h after transfection, cells were treated with 15 μM MG-132 for 0, 3, 6, and 12 h, respectively. After treatment, cells were fixed in 4% paraformaldehyde (Solarbio; P1110) for 20 min and stained with DAPI (300 nM; Thermo Fisher Scientific) at room temperature for 20 min. Cells were rinsed with PBS and sealed in antifade mounting Medium (Beyotime; P0126). Results were analyzed by using SP8 microscope (Leica Microsystems, Germany).

### Quantitative RT-PCR

The RNA was isolated from cells using TRIzol reagent (Invitrogen, United States). First- strand cDNA was synthesized by using FastQuant RT Kit (TIANGEN) according to the manufacturer’s instructions. The newly synthesized cDNA was amplified in a 20 μL reaction by using SYBR green kit (TIANGEN). The reaction was detected by QuantStudio 5 Real-Time PCR System (Thermo Fisher Scientific). The following primers were used to amplify the specific genes: LC3 (Forward: 5′-AGCAGCATCCAACCAAAATC-3′; Reverse: 5′-TGTGTCCG TTCACCAACAG-3′), LAMP-1 (Forward: 5′-CTGCCTTTAAA GCTGCCAAC-3′; Reverse: 5′-TGTTCTCGTCCAGCAGACAC-3′), cathepsin D (Forward: 5′-CTTCGACAACCTGATGCAGC-3′; Reverse: 5′-TACTTGGAGTCTGTGCCACC-3′), β-actin (Forward: 5′-CCTGGCACCCAGCACAAT-3′; Reverse: 5′-GGG CCGGACTCGTCATAC-3′). Amplification conditions were as follows: initial denaturation at 95°C for 15 min, followed by 40 cycles comprising denaturation at 95°C for 10 s, annealing at 60°C for 20 s, and extension at 72°C for 30 s.

### Semi-Quantitative RT-PCR

The newly synthesized cDNA was amplified in a 10 μL reaction. The following primers were used to amplify the specific genes: LC3 (Forward: 5′-AGCAGCATCCAACCAAAATC-3′; Reverse: 5′-TGTGTCCGTTCACCAACAG-3′), LAMP-1 (Forward: 5′-CTGCCTTTAAAGCTGCCAAC-3′; Reverse: 5′-TGTTCTCGTCCAGCAGACAC-3′), and β-actin (Forward: 5′-CCTGGCACCCAGCACAAT-3′; Reverse: 5′-GGGCCGG ACTCGTCATAC-3′). Amplification conditions were as follows: initial denaturation at 95°C for 3 min, followed by 25–35 cycles comprising denaturation at 95°C for 30 s, annealing at the optimized temperature for each set of primers for 15 s, and extension at 72°C for 45 s. The final extension was carried out at 72°C for 5 min. The products were analyzed on a 1.0% agarose gel prepared in TAE buffer.

### Data Analysis

Quantifications were performed with three or more independent experiments. Values represent mean ± SEM. The data was analyzed by one-way ANOVA followed by Tukey’s test or Student’s *t*-test. *P* < 0.05 was considered to be statistically significant.

## Results

### The Half-Life of TFEB Is Approximately 13.5 h in SH-SY5Y Cells

**FIGURE 1:**
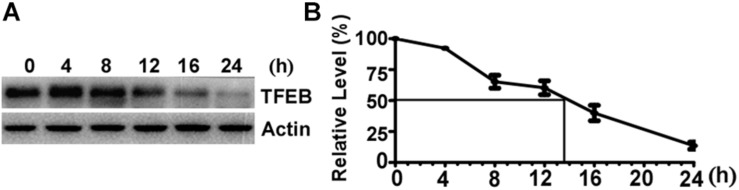
The half-life of Transcription Factor EB is approximately 13.5 h in SH-SY5Y cells. **(A)** SH-SY5Y cells were treated with 100 μg/mL cycloheximide and were harvested at 0, 4, 8, 12, 16, and 24 h after drug treatment. Cell lysates were separated on 10% SDS-PAGE. TFEB antibody was used to detect TFEB. Actin was detected by its antibody and used as the internal control. **(B)** TFEB levels were plotted as a percentage of the control (0 h). Values are mean ± SEM; *n* ≥ 3.

To determine the turnover rate of TFEB protein in SH-SY5Y cells, 100 μg/mL CHX was applied to halt protein synthesis by blocking the translation of messenger RNA. The cells were harvested at 0 (control), 4, 8, 12, 16, and 24 h after CHX treatment, respectively. Western blot was performed to determine the amount of TFEB in control cells and CHX treated cells (Figure 1A). The relative amount of remaining TFEB in CHX treated cells was calculated according to the amount of TFEB in control cells. The level of TFEB was reduced to 60.36 and 13.54% of the control after CHX treatment for 12 and 24 h, respectively (Figure 1B). Based on the curve, the TFEB reduced to 50% at 13.5 h after CHX treatment. It indicated that the half-life of TFEB is approximately 13.5 h in SH-SY5Y cells.

### Proteasome Inhibition Promotes TFEB Accumulation and Dephosphorylation

**FIGURE 2:**
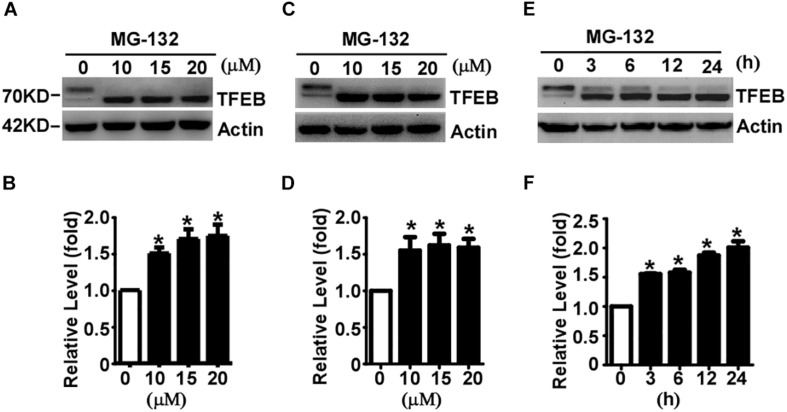
Proteasome inhibition facilitates TFEB accumulation and dephosphorylation. SH-SY5Y cells **(A)** or HEK293 cells **(C)** were treated with MG-132 at indicated dosage. Whole-cell lysates were separated by 10% SDS-PAGE. TFEB was detected by TFEB antibody. Actin served as a loading control. **(B,D)** Quantification of TFEB shown in **(A,C)**, respectively. **(E)** HEK293 cells were treated with 15 μM MG-132 for indicated time course. **(F)** Quantification of TFEB shown in panel **(E)**. Values are mean ± SEM; *n* ≥ 3, ^∗^*P* < 0.05 by one-way ANOVA followed by Tukey’s test.

It has been reported that TFEB is degraded by UPS in Hela cells (Sha et al., 2017). To determine whether TFEB is degraded via the UPS in neuronal-like cells, proteasome inhibitor MG-132 was applied to SH-SY5Y cells for 24 h at the doses of 0 (control), 10, 15, and 20 μM, respectively. The efficacy of MG-132 has been validated by its inhibitory effect on TMP21 degradation (Supplementary Figure S1), which has been reported previously (Liu et al., 2008). Compared with that in control cells, total TFEB levels were significantly increased to 1.51 ± 0.08, 1.70 ± 0.13, and 1.75 ± 0.15 fold, respectively (Figures 2A,B). To confirm the effect of MG-132 on TFEB accumulation is not a cell type specific effect, same experiment was performed in HEK293 cells. Consistently, MG-132 significantly increases total TFEB expression to 1.55 ± 0.18, 1.62 ± 0.16, and 1.59 ± 0.12 fold at the doses of 10, 15, and 20 μM, respectively (Figures 2C,D). In time-course experiments, HEK293 cells were treated with 15 μM MG-132 for 0, 3, 6, 12, and 24 h, respectively. The levels of TFEB were increased to 1.56 ± 0.02, 1.58 ± 0.08, 1.87 ± 0.08, and 2.00 ± 0.19 fold, respectively (Figures 2E,F).

The phosphorylated TFEB (high molecular weight) and dephosphorylated TFEB (low molecular weight) were detected in control cells although dephosphorylated TFEB is much less than phosphorylated TFEB (Figures 2A,C,E). MG-132 treatment significantly shifted TFEB from phosphorylated form to dephosphorylated form (Figures 2A,C,E). In both SH-SY5Y and HEK293 cells, 24-hour MG-132 treatment completely shifts phosphorylated TFEB to dephosphorylated form at the dosage of 10, 15, and 20 μM, respectively (Figures 2A,C). In time-course experiments, phosphorylated TFEB gradually shifts to the dephosphorylated form in a time-dependent manner (Figure 2E). Our data demonstrated that proteasome inhibition not only promotes TFEB accumulation but also shifts TFEB to the dephosphorylated form, indicating that TFEB is degraded by proteasome pathway and proteasome inhibition promotes TFEB dephosphorylation.

### Proteasome Inhibition Facilitates TFEB Nuclear Translocation

**FIGURE 3:**
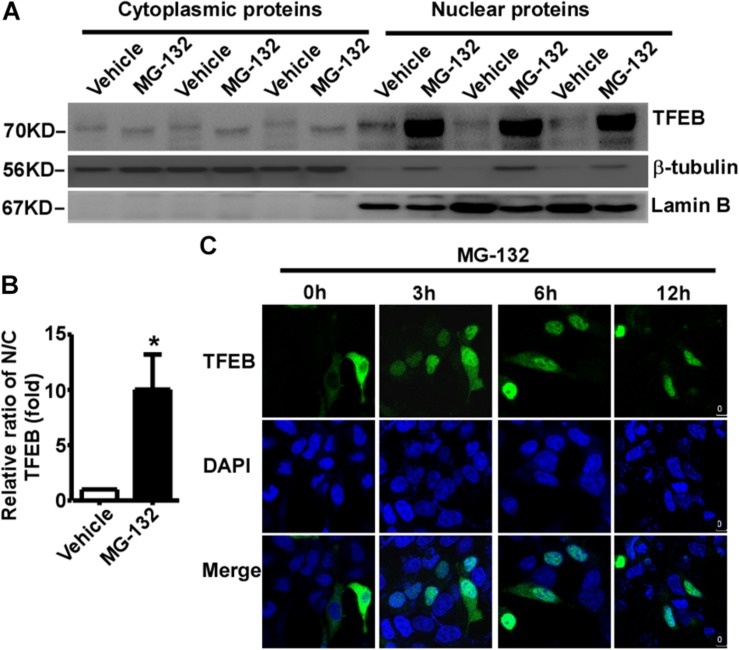
Proteasome inhibition promotes TFEB nuclear translocation. **(A)** HEK293 cells were treated with 15 μM MG-132 for 24 h. The cell lysates were fractionated into cytoplasmic and nuclear fractions. Both nuclear and cytoplasmic fractions were separated by 10% SDS-PAGE. TFEB was detected by TFEB antibody. β-tubulin was detected by β-tubulin antibody and used as the control of cytoplasmic fraction. Lamin B was detected by lamin B antibody and used as the control of nuclear fraction. **(B)** Relative ratio of nuclear TFEB to cytoplasmic TFEB. N, nuclear; C, cytoplasmic. Values are mean ± SEM; *n* ≥ 3, ^∗^*P* < 0.05 by Student’s *t*-test. **(C)** TFEB-GFP was transfected into HEK293 cells. 24 h after transfection, cells were treated with 15 μM MG-132 for 0, 3, 6, and 12 h, respectively. After fixation, cells were stained with DAPI. Confocal microscope was used to analyze the results. Nuclei were stained with DAPI (blue).

The subcellular distribution of TFEB mainly depends on its phosphorylation status. Phosphorylated TFEB is mainly located in the cytoplasm, while dephosphorylated TFEB mainly translocates to the nucleus. Our data showed that proteasome inhibition promotes TFEB dephosphorylation, suggesting that proteasome inhibition might promote TFEB nuclear translocation. To confirm this effect, cytoplasmic and nuclear proteins were extracted from control cells and MG-132 treated cells. 24 h after treatment with vehicle or 15 μM MG-132, cytoplasmic and nuclear proteins were extracted and analyzed. No nuclear protein lamin B was detected in the cytoplasmic fraction, while cytoplasmic protein β-tubulin was detected (Figure 3A). As expected, phosphorylated TFEB was detected in the cytoplasmic fraction of control cells, while dephosphorylated TFEB was detected in the MG-132 treated cells although the signal is weak (Figure 3A). In the nuclear fraction, abundant lamin B was detected and extremely weak signal of cytoplasmic protein β-tubulin was detected (Figure 3A). As expected, the large amount of dephosphorylated TFEB was detected in the nuclear fraction of MG-132 treated cells but not in control cells. In MG-132 treated cells, the ratio of nuclear TFEB to cytoplasmic TFEB was significantly increased to 10.03 ± 3.18 fold (Figure 3B). It indicated that proteasome inhibition promotes nuclear translocation of dephosphorylated TFEB.

To further confirm proteasome inhibition-induced TFEB nuclear translocation, GFP tagged TFEB was transfected into HEK293 cells. 24 h after transfection, 15 μM MG-132 was applied. TFEB was mainly located in the cytoplasm in controls cells (0 h), while MG-132 treatment promoted nuclear translocation of TFEB at 3, 6, and 12 h time points, respectively (Figure 3C). The data indicated that proteasome inhibition promotes nuclear translocation of TFEB.

### Proteasome Inhibition Facilitates TFEB-Mediated ALP Activation

**FIGURE 4:**
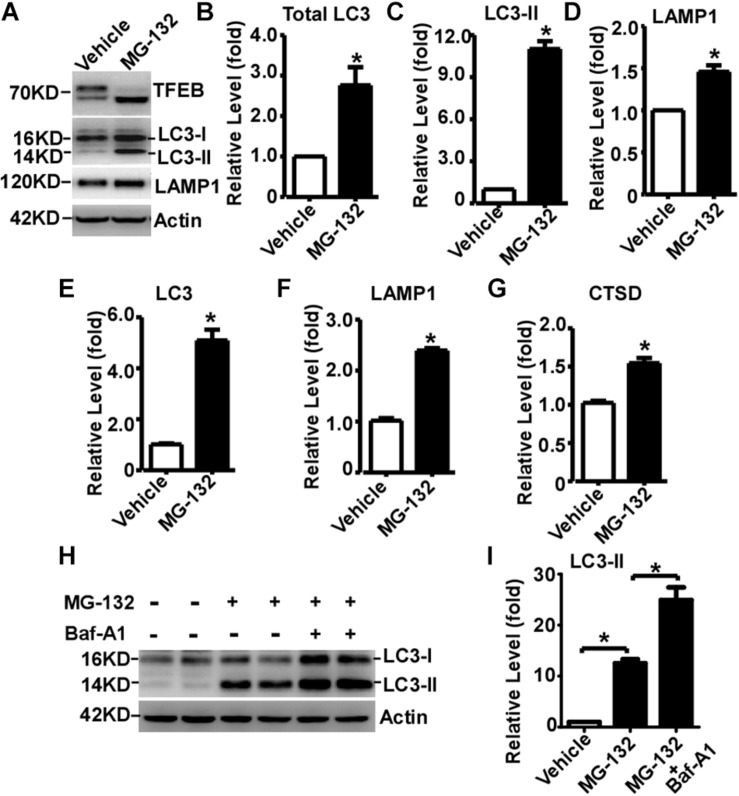
Proteasome inhibition facilitates TFEB-mediated autophagy- lysosome pathway activation. **(A)** HEK293 cells were treated with 15 μM MG-132 for 24 h. Whole-cell lysates were separated by 12% SDS-PAGE. TFEB was detected by TFEB antibody. LAMP-1 was detected by LAMP-1 antibody. LC3-I and LC3-II were detected by LC3 antibody. Actin served as a loading control. **(B–D)** Quantification of total LC3, LC3-II and LAMP-1 expression. HEK293 cells were treated with 15 μM MG-132 for 24 h. The mRNA levels of LC3, LAMP1 and cathepsin D (CTSD) were examined by qRT-PCR, shown in **(E–G)**, respectively. **(H)** HEK293 cells were treated with 15 μM MG-132 for 24 h, and then treated with 300 nM Bafilomycin A1 (Baf-A1) for 4 h. Whole-cell lysates were separated by 12% SDS-PAGE. LC3-I and LC3-II were detected by LC3 antibody. Actin served as a loading control. **(I)** Quantification of LC3-II. Values are mean ± SEM; *n* ≥ 3, ^∗^*P* < 0.05 by Student’s *t*-test or by one-way ANOVA followed by Tukey’s test.

Transcription Factor EB is activated by its dephosphorylation and nuclear translocation. To examine whether proteasome inhibition-induced TFEB dephosphorylation and nuclear translocation promotes ALP activation, two downstream targets of TFEB were examined. LC3-II is the marker of autophagosome, while LAMP1 is the marker of lysosome. Total LC3 was significantly increased to 2.76 ± 0.44 fold in MG-132 treated cells compared with that in control cells, while LC3-II was markedly increased to 11.01 ± 0.56 fold (Figures 4A–C). Moreover, LAMP1 was dramatically increased to 1.46 ± 0.08 fold in MG-132 treated cells compared with that in control cells (Figures 4A,D). To explore whether proteasome inhibition-induced TFEB nuclear translocation promotes the transcription of its downstream targets for the biogenesis of autophagosome and lysosome, the mRNA levels of LC3, LAMP1 and cathepsin D were examined by qRT-PCR. The mRNA levels of LC3, LAMP1 and cathepsin D were significantly increased to 5.10 ± 0.42, 2.39 ± 0.05, and 1.55 ± 0.07 fold, respectively (Figures 4E–G). Consistently, semi-quantitative RT-PCR data also showed that the mRNA levels of LC3 and LAMP1 were significantly increased (Supplementary Figure S2).

To further investigate that the increase of LC3-II is caused by autophagic flux impairment or increased autophagosome biogenesis, Baf-A1, an inhibitor blocking autophagosome-lysosome fusion, was applied with MG-132. MG-132 significantly increases the level of LC3-II to 12.53 ± 0.78 fold, while Baf-A1 along with MG-132 further increases the level of LC3-II to 24.93 ± 2.47 fold (Figures 4H,I). It demonstrated that proteasome inhibition-induced LC3-II elevation is mediated by increased LC3-II expression but not by the blockade of LC3-II degradation, indicating that proteasome inhibition increases autophagosome biogenesis but not impairs autophagic flux. As short-term (only 3h) MG-132 treatment is enough to initiate irreversible effect on cells, the reversible effect of MG-132 withdrawal on ALP activation has not been examined (Supplementary Figure S3).

## Discussion

Sha et al. reported that TFEB is degraded by proteasome pathway with a half-life of 4–6 h in Hela cells (Sha et al., 2017). The turnover rate of TFEB in neuronal-like cells has not been characterized although TFEB is highly expressed in CNS and plays a pivotal role in the maintenance of CNS functions. Here we showed that the half-life of TFEB in SH-SY5Y cells is approximately 13.5 h, suggesting that the degradation rate is lower in SH-SY5Y cells than that in Hela cells. However, it has to be noted that the half-life measurement by S^35^-labeled pulse-chase strategy has to go through immunoprecipitation, which might contribute to the difference. Consistent with the previous study, we showed that TFEB is degraded by the proteasome pathway in both SH-SY5Y and HEK293. It suggested that the proteasome pathway is a major pathway for TFEB degradation without cell type difference.

Although proteasome inhibition-induced ALP activation is well documented, underlying mechanisms remain elusive. Our data showed that proteasome inhibition not only promotes TFEB accumulation but also facilitates TFEB dephosphorylation. It has been reported that proteasome inhibition activates calcineurin which is a major phosphatase contributing to the dephosphorylation of TFEB (Medina et al., 2015). Thus, proteasome inhibition-induced TFEB dephosphorylation might be caused by calcineurin activation. Moreover, phosphorylated TFEB is mainly located in the cytoplasm, while TFEB dephosphorylation facilitates it translocation to the nucleus. Consistently, our data showed that proteasome inhibition-induced TFEB accumulation mainly translocates into the nucleus. Nuclear TFEB binds to the CLEAR element within the promoter region of LC3 and LAMP1 genes contributing to the upregulation of their transcription. As expected, increased levels of LC3, cathepsin D, and LAMP1 mRNA were observed in MG-132 treated cells along with the nuclear translocation of TFEB. It highly indicated that TFEB is key mediator in proteasome inhibition-induced ALP activation.

Our data showed that TFEB is a key mediator in proteasome inhibition-induced ALP activation. The impairment of proteasome function is well documented in neurodegenerative diseases, while both the activation of TFEB/ALP and inactivation of TFEB/ALP has been reported in neurodegenerative diseases (Martini-Stoica et al., 2016; Finkbeiner, 2019). Our data indicated that TFEB might play a key role in proteasome impairment-induced ALP activation in neurodegenerative diseases. However, it seems to be in conflict with the fact that proteasome impairment along with the deficiency of TFEB/ALP in neurodegenerative diseases. It has to be noted that the bidirectional alteration of TFEB/ALP might be associated with the stages of diseases, duration of the stress and other regulation pathways. Our data showed that short-term proteasome inhibition significantly activates TFEB-mediated ALP, which might explain the mechanism of TFEB/ALP activation concomitant with proteasome impairment at the early stage of neurodegenerative diseases. However, long-term proteasome impairment might lead to ALP inhibition via various mechanisms. First, compensatory activation of ALP might be exhausted by long-term stress conditions, resulting in ALP impairment. Moreover, other regulation pathways might also contribute to the inhibition of ALP (Cortes and La Spada, 2019). For example, increased activity of GSK3β in AD has been reported in many studies, which might contribute to ALP inhibition by promoting TFEB phosphorylation and subsequent cytoplasmic retention (Ly et al., 2013; Li et al., 2016).

## Conclusion

In conclusion, the half-life TFEB is approximately 13.5 h in neuronal-like cells, and TFEB is degraded through proteasome pathway in both neuronal-like and non-neuronal cells. Moreover, proteasome impairment not only promotes TFEB accumulation but also facilitates its dephosphorylation and nuclear translocation. Furthermore, proteasome inhibition-induced TFEB accumulation, dephosphorylation and nuclear translocation significantly increases the expression of a number of TFEB downstream genes involved in ALP activation, including LC3, particularly LC3-II, cathepsin D and LAMP1. Furthermore, we demonstrated that proteasome inhibition increases autophagosome biogenesis but not impairs autophagic flux.

## Data Availability

The raw data supporting the conclusions of this manuscript will be made available by the authors, without undue reservation, to any qualified researcher. The uncropped Western blot images can be found in the Supplementary Material.

## Author Contributions

YW conceived and designed the study. CL, XW, XL, KQ, and FJ performed the experiments. CL, XW, YiL, QK, and YaL analyzed the data. CL and YW wrote the manuscript. CL, XW, KQ, FJ, YaL, and YW revised the manuscript.

## Conflict of Interest Statement

The authors declare that the research was conducted in the absence of any commercial or financial relationships that could be construed as a potential conflict of interest.

## Supplementary Material

FIGURE S1 Proteasome inhibition facilitates TMP21 accumulation. HEK293 cells were treated with 15 μM MG-132 for indicated time course. Whole-cell lysates were separated by 10% SDS-PAGE. TMP21 was detected by TMP21 antibody. Actin served as a loading control.

FIGURE S2 Proteasome inhibition facilitates TFEB-mediated ALP activation. **(A)** HEK293 cells were treated with 15 μM MG-132 for 24 h. Total RNA was extracted and followed by RT-PCR. PCR products of LC3, LAMP1 and actin were resolved on 1.0% agarose gel, respectively. **(B,C)** Quantification of LC3 and LAMP1 mRNA levels. Values are mean ± SEM; *n* ≥ 3, ^∗^*P* < 0.05 by Student’s *t*-test.

FIGURE S3 MG-132 withdrawal has no reversible effect on HEK293 cells. HEK293 cells were cultured in media with or without 15 μM MG-132 (control or MG-132 3 h). After 3 h MG-132 treatment, the culture media were replaced with the fresh media without MG-132 for indicated recovery time course. HEK293 cells were examined under light microscope at X100 view.

DATA SHEET S1 All western blots without being cropped in Figures 1–4, *n* >= 3.
