# Supplementary figures and images for: Proteasome Inhibition Activates Autophagy-Lysosome Pathway Associated With TFEB Dephosphorylation and Nuclear Translocation
Source: Front Cell Dev Biol. 2019 Aug 22;7:170. doi: 10.3389/fcell.2019.00170 (PMC6713995; doi:10.3389/fcell.2019.00170)

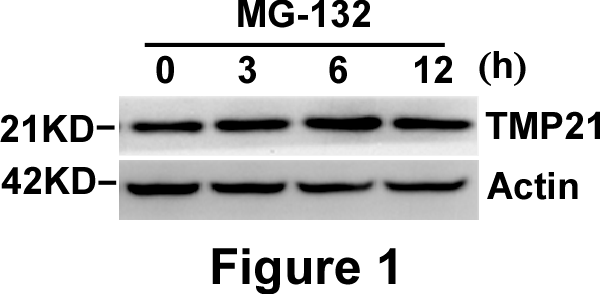

Supplement: FIGURE S1 — Proteasome inhibition facilitates TMP21 accumulation. HEK293 cells were treated with 15 μM MG-132 for indicated time course. Whole-cell lysates were separated by 10% SDS-PAGE. TMP21 was detected by TMP21 antibody. Actin served as a loading control. [file Image_1.TIF]

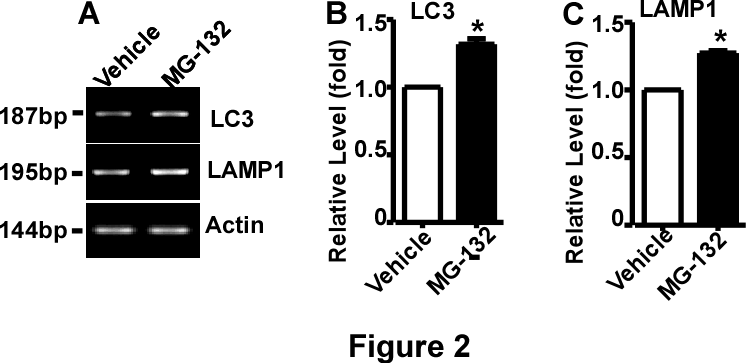

Supplement: FIGURE S2 — Proteasome inhibition facilitates TFEB-mediated ALP activation. (A) HEK293 cells were treated with 15 μM MG-132 for 24 h. Total RNA was extracted and followed by RT-PCR. PCR products of LC3, LAMP1 and actin were resolved on 1.0% agarose gel, respectively. (B,C) Quantification of LC3 and LAMP1 mRNA levels. Values are mean ± SEM; n ≥ 3, ∗P < 0.05 by Student’s t-test. [file Image_2.TIF]

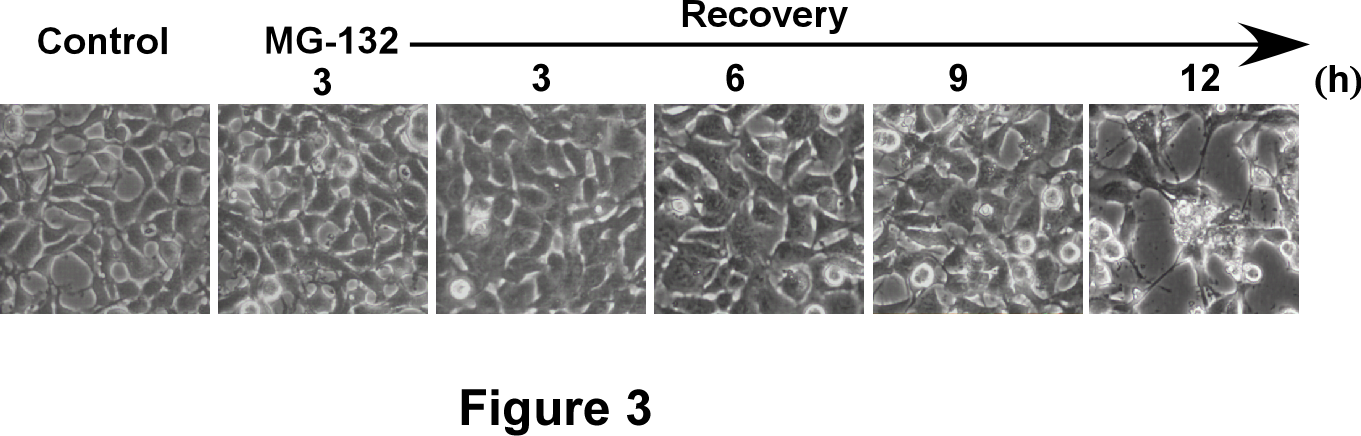

Supplement: FIGURE S3 — MG-132 withdrawal has no reversible effect on HEK293 cells. HEK293 cells were cultured in media with or without 15 μM MG-132 (control or MG-132 3 h). After 3 h MG-132 treatment, the culture media were replaced with the fresh media without MG-132 for indicated recovery time course. HEK293 cells were examined under light microscope at X100 view. [file Image_3.tif]

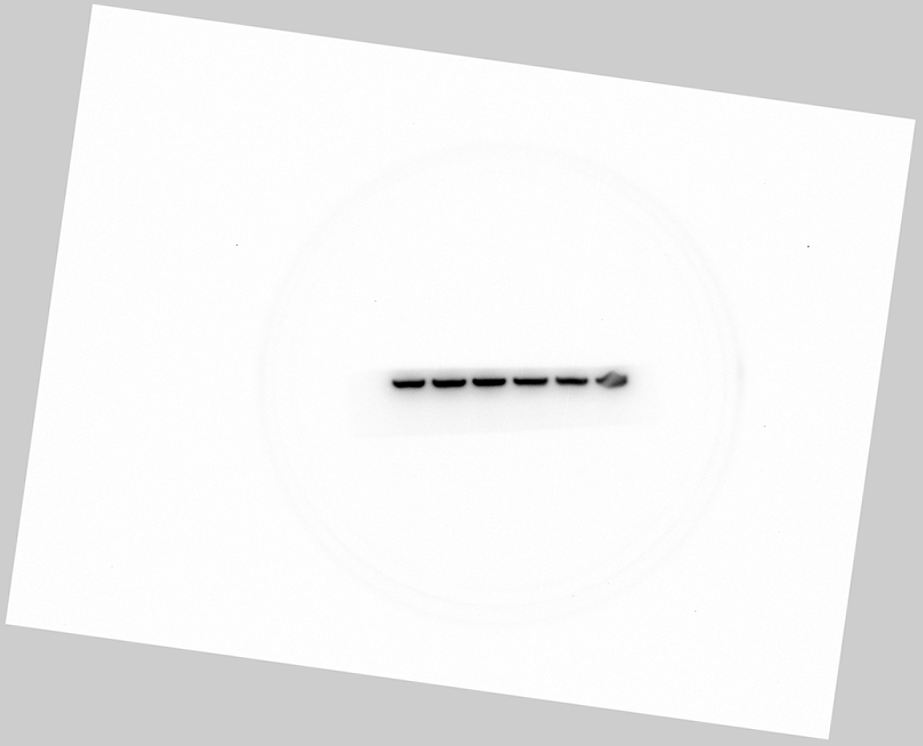

Supplement: DATA SHEET S1 — All western blots without being cropped in Figures 1–4, n >= 3. [file Data_Sheet_1.ZIP › full images of western blots/figure 1/1/actin.tif]

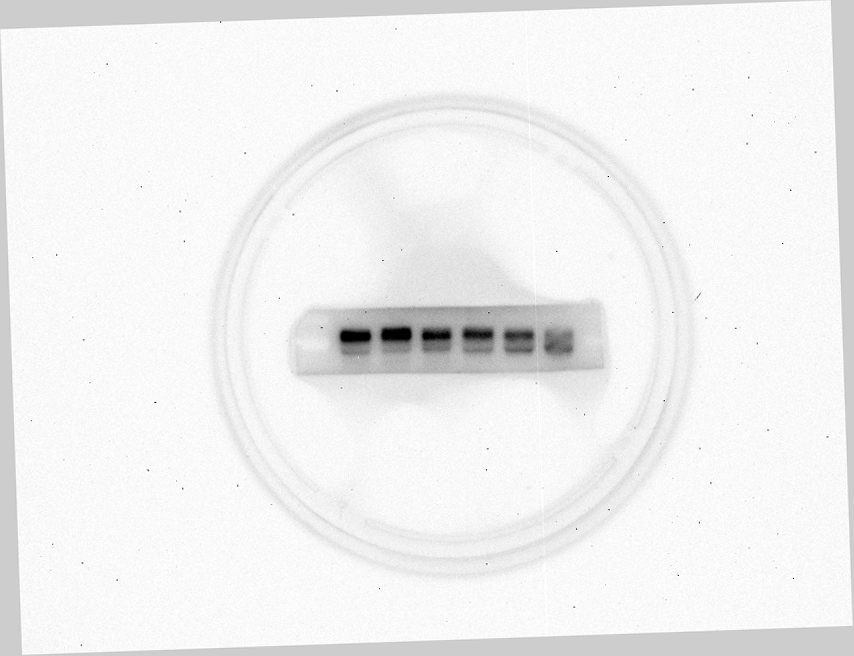

Supplement: DATA SHEET S1 — All western blots without being cropped in Figures 1–4, n >= 3. [file Data_Sheet_1.ZIP › full images of western blots/figure 1/1/TFEB.tif]

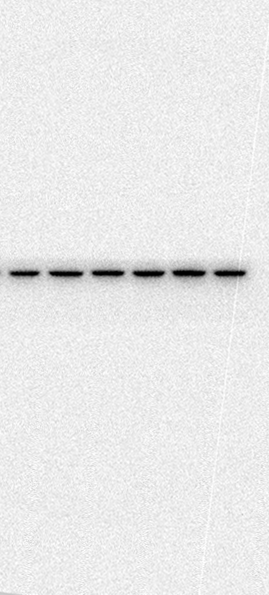

Supplement: DATA SHEET S1 — All western blots without being cropped in Figures 1–4, n >= 3. [file Data_Sheet_1.ZIP › full images of western blots/figure 1/2/actin.tif]

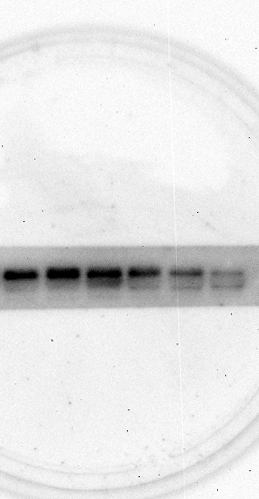

Supplement: DATA SHEET S1 — All western blots without being cropped in Figures 1–4, n >= 3. [file Data_Sheet_1.ZIP › full images of western blots/figure 1/2/TFEB.tif]

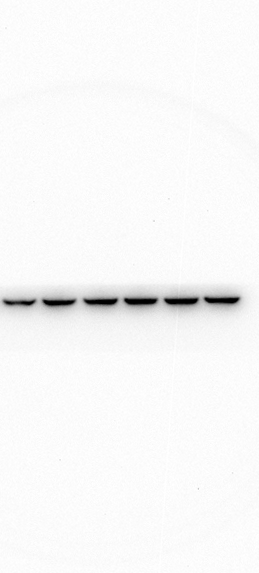

Supplement: DATA SHEET S1 — All western blots without being cropped in Figures 1–4, n >= 3. [file Data_Sheet_1.ZIP › full images of western blots/figure 1/3/actin.tif]

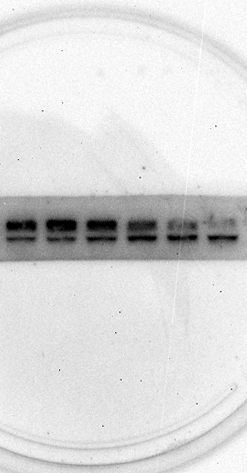

Supplement: DATA SHEET S1 — All western blots without being cropped in Figures 1–4, n >= 3. [file Data_Sheet_1.ZIP › full images of western blots/figure 1/3/TFEB.tif]

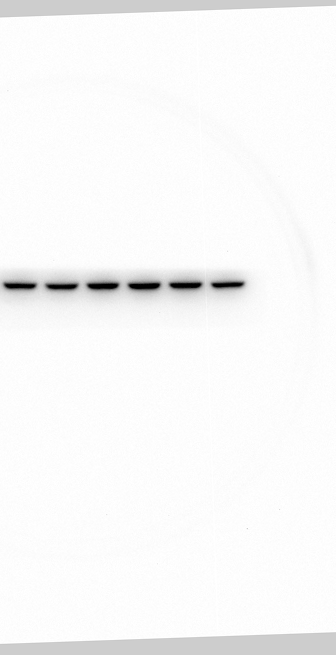

Supplement: DATA SHEET S1 — All western blots without being cropped in Figures 1–4, n >= 3. [file Data_Sheet_1.ZIP › full images of western blots/figure 1/4/actin.tif]

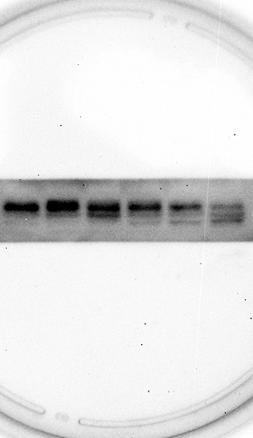

Supplement: DATA SHEET S1 — All western blots without being cropped in Figures 1–4, n >= 3. [file Data_Sheet_1.ZIP › full images of western blots/figure 1/4/TFEB.tif]

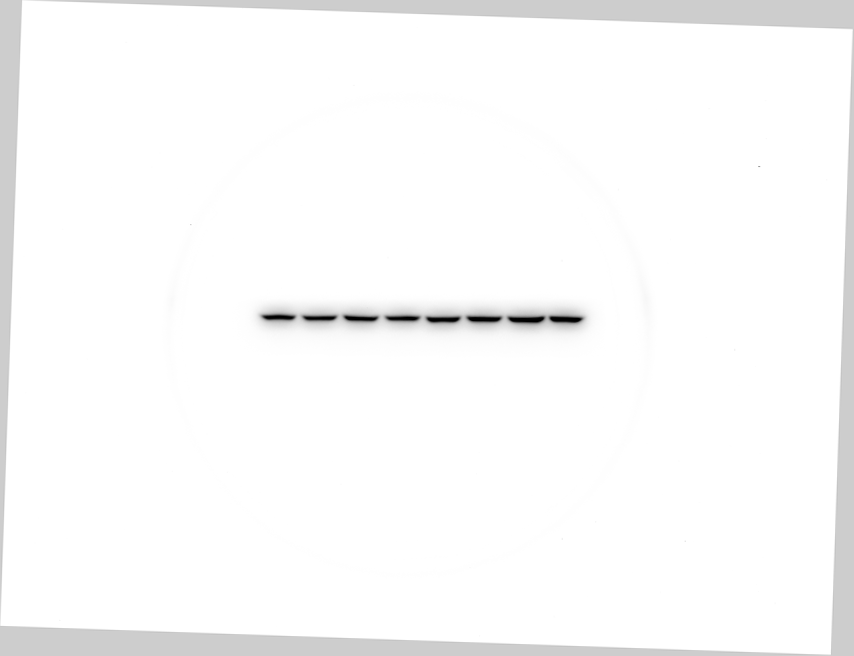

Supplement: DATA SHEET S1 — All western blots without being cropped in Figures 1–4, n >= 3. [file Data_Sheet_1.ZIP › full images of western blots/figure 2/A/1-2/actin.tif]

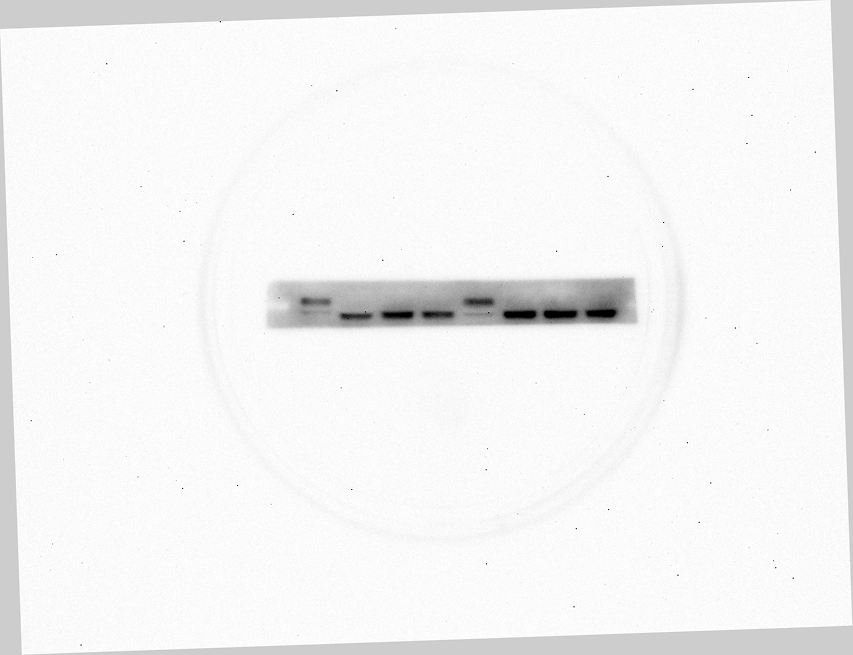

Supplement: DATA SHEET S1 — All western blots without being cropped in Figures 1–4, n >= 3. [file Data_Sheet_1.ZIP › full images of western blots/figure 2/A/1-2/TFEB.tif]

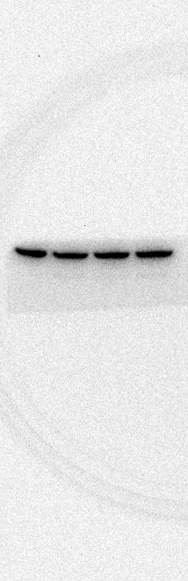

Supplement: DATA SHEET S1 — All western blots without being cropped in Figures 1–4, n >= 3. [file Data_Sheet_1.ZIP › full images of western blots/figure 2/A/3/actin.tif]

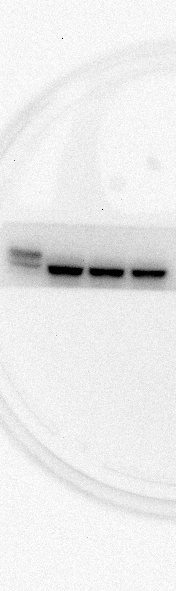

Supplement: DATA SHEET S1 — All western blots without being cropped in Figures 1–4, n >= 3. [file Data_Sheet_1.ZIP › full images of western blots/figure 2/A/3/TFEB.tif]

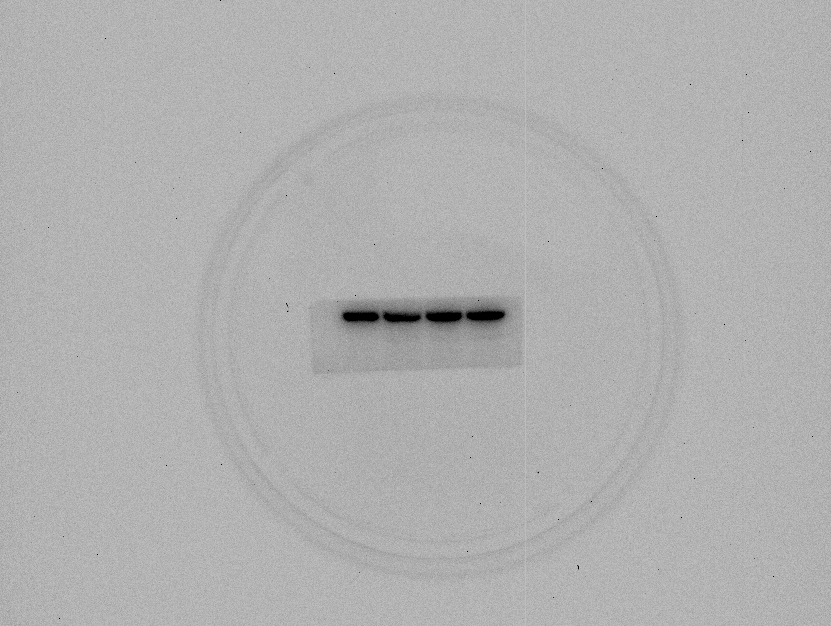

Supplement: DATA SHEET S1 — All western blots without being cropped in Figures 1–4, n >= 3. [file Data_Sheet_1.ZIP › full images of western blots/figure 2/A/4/actin.tif]

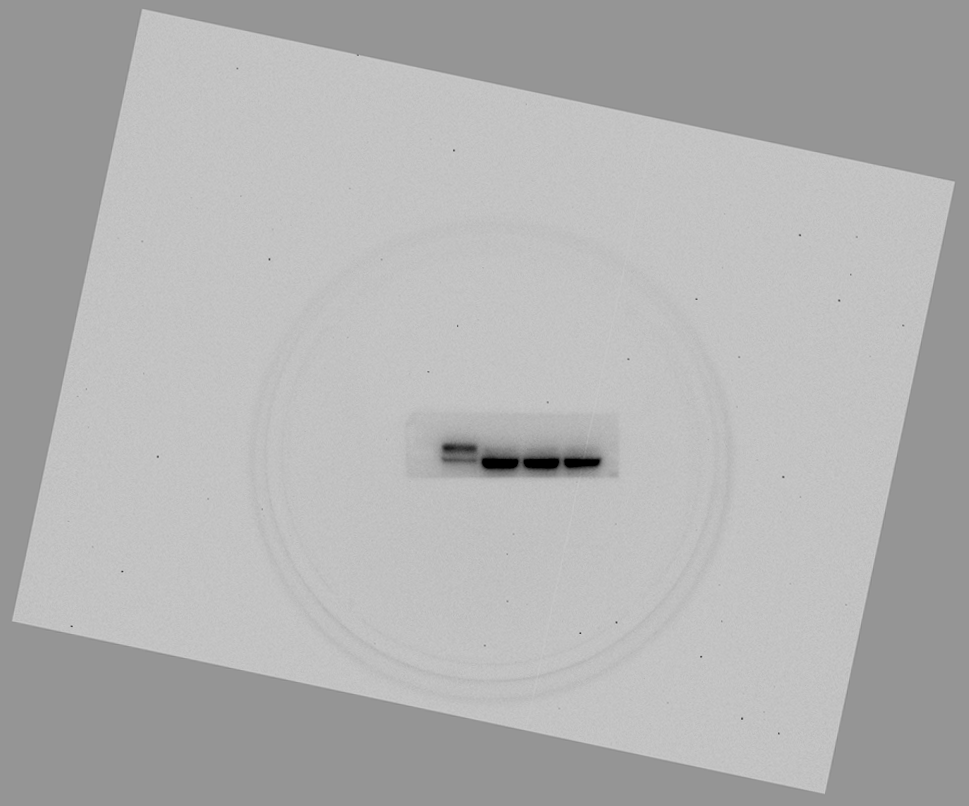

Supplement: DATA SHEET S1 — All western blots without being cropped in Figures 1–4, n >= 3. [file Data_Sheet_1.ZIP › full images of western blots/figure 2/A/4/TFEB.tif]

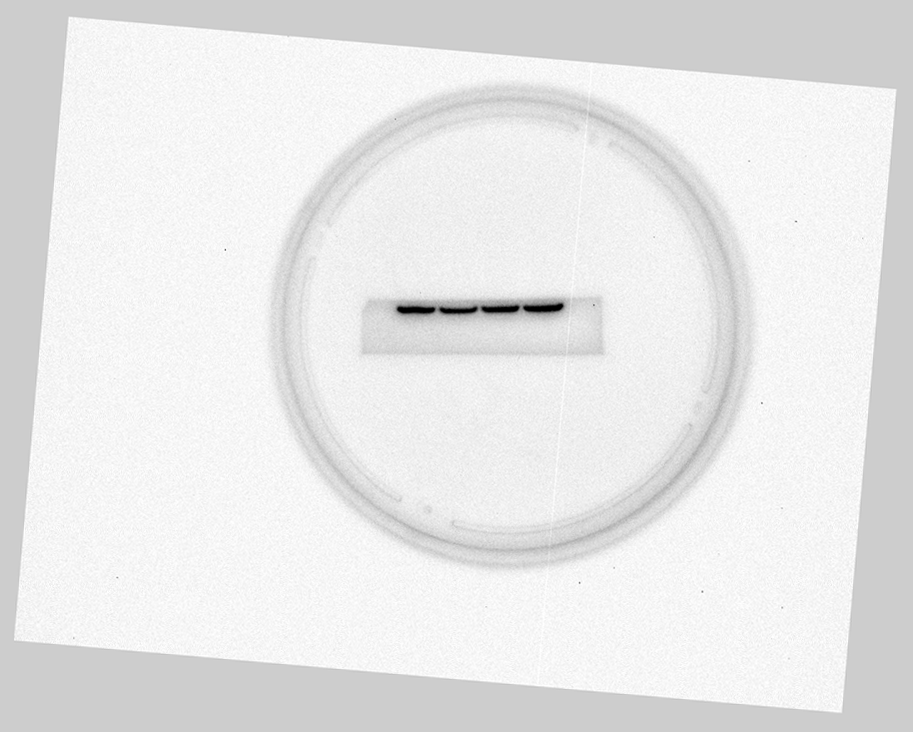

Supplement: DATA SHEET S1 — All western blots without being cropped in Figures 1–4, n >= 3. [file Data_Sheet_1.ZIP › full images of western blots/figure 2/C/1/actin.tif]

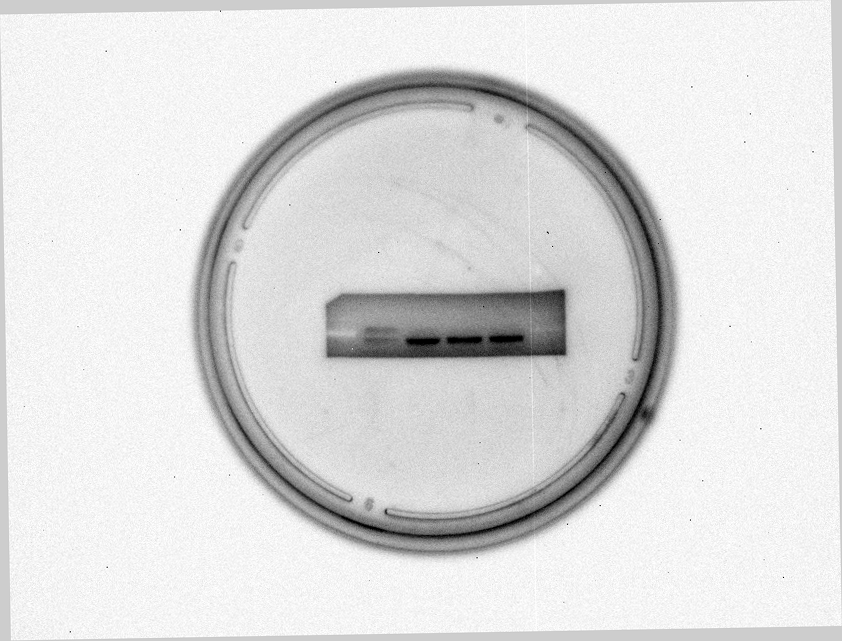

Supplement: DATA SHEET S1 — All western blots without being cropped in Figures 1–4, n >= 3. [file Data_Sheet_1.ZIP › full images of western blots/figure 2/C/1/TFEB.tif]

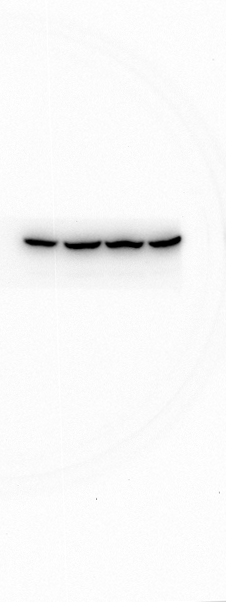

Supplement: DATA SHEET S1 — All western blots without being cropped in Figures 1–4, n >= 3. [file Data_Sheet_1.ZIP › full images of western blots/figure 2/C/2/actin.tif]

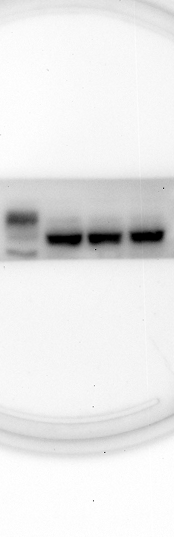

Supplement: DATA SHEET S1 — All western blots without being cropped in Figures 1–4, n >= 3. [file Data_Sheet_1.ZIP › full images of western blots/figure 2/C/2/TFEB.tif]

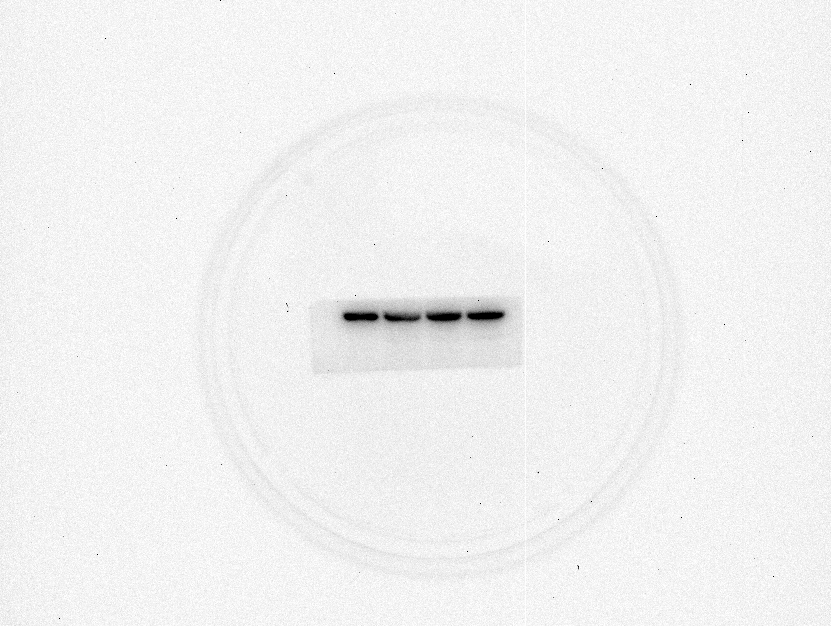

Supplement: DATA SHEET S1 — All western blots without being cropped in Figures 1–4, n >= 3. [file Data_Sheet_1.ZIP › full images of western blots/figure 2/C/3/actin.tif]

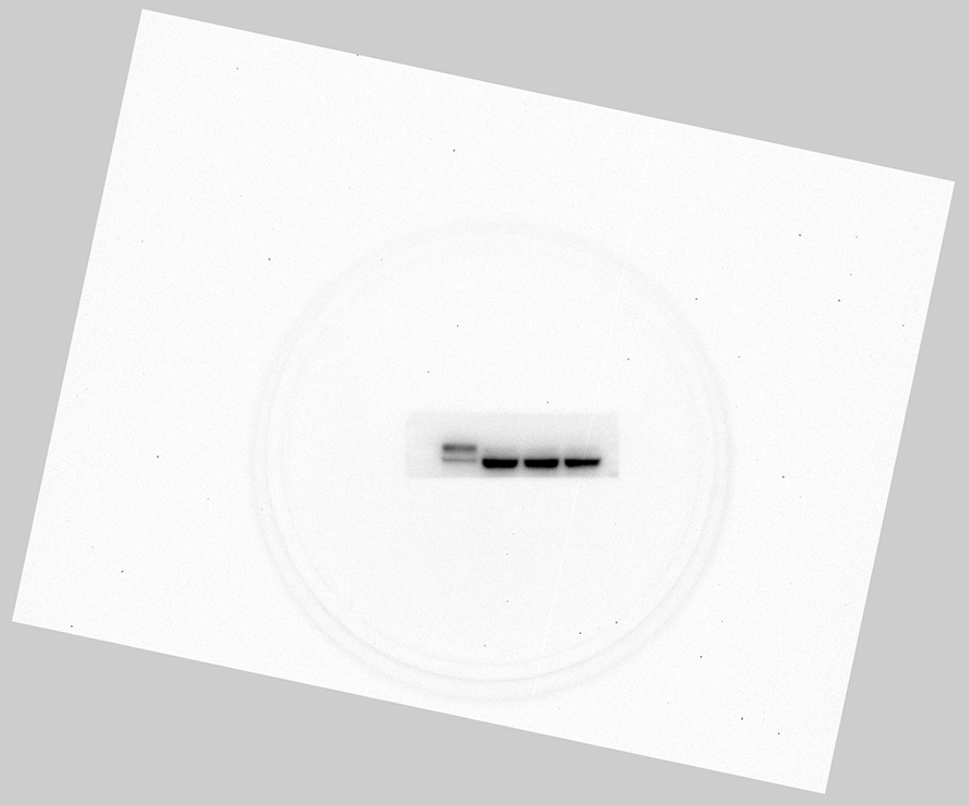

Supplement: DATA SHEET S1 — All western blots without being cropped in Figures 1–4, n >= 3. [file Data_Sheet_1.ZIP › full images of western blots/figure 2/C/3/TFEB.tif]

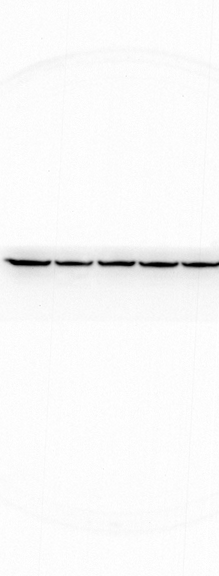

Supplement: DATA SHEET S1 — All western blots without being cropped in Figures 1–4, n >= 3. [file Data_Sheet_1.ZIP › full images of western blots/figure 2/E/1/actin.tif]

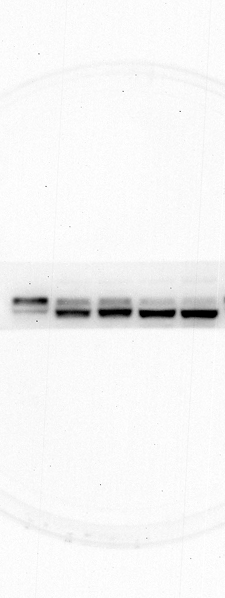

Supplement: DATA SHEET S1 — All western blots without being cropped in Figures 1–4, n >= 3. [file Data_Sheet_1.ZIP › full images of western blots/figure 2/E/1/TFEB.tif]

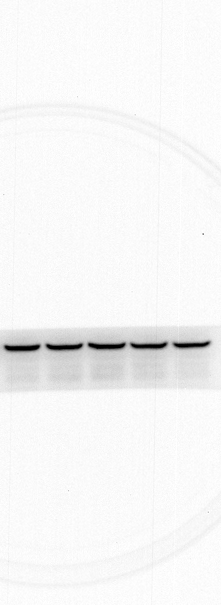

Supplement: DATA SHEET S1 — All western blots without being cropped in Figures 1–4, n >= 3. [file Data_Sheet_1.ZIP › full images of western blots/figure 2/E/2/actin.tif]

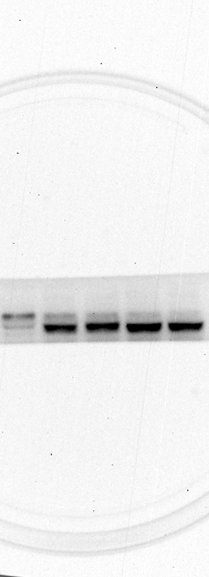

Supplement: DATA SHEET S1 — All western blots without being cropped in Figures 1–4, n >= 3. [file Data_Sheet_1.ZIP › full images of western blots/figure 2/E/2/TFEB.tif]

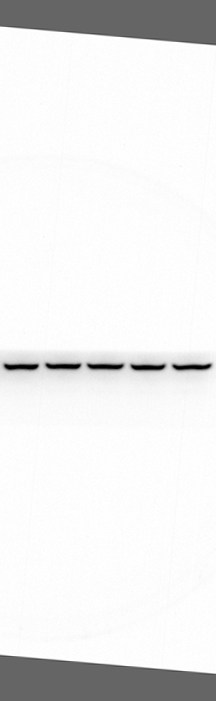

Supplement: DATA SHEET S1 — All western blots without being cropped in Figures 1–4, n >= 3. [file Data_Sheet_1.ZIP › full images of western blots/figure 2/E/3/actin.tif]

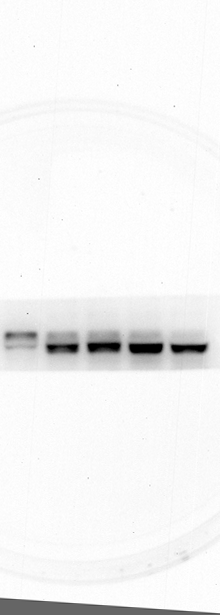

Supplement: DATA SHEET S1 — All western blots without being cropped in Figures 1–4, n >= 3. [file Data_Sheet_1.ZIP › full images of western blots/figure 2/E/3/TFEB.tif]

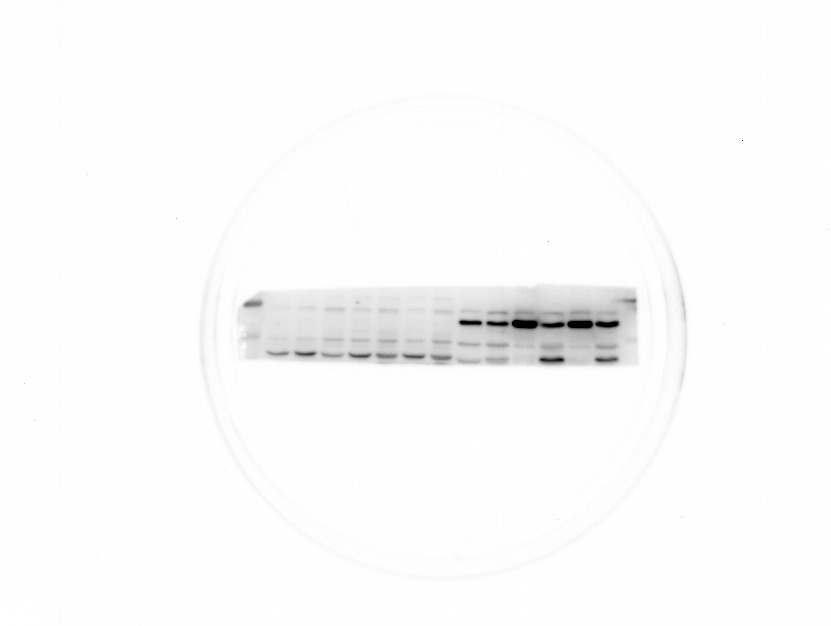

Supplement: DATA SHEET S1 — All western blots without being cropped in Figures 1–4, n >= 3. [file Data_Sheet_1.ZIP › full images of western blots/figure 3/Lamin B.tif]

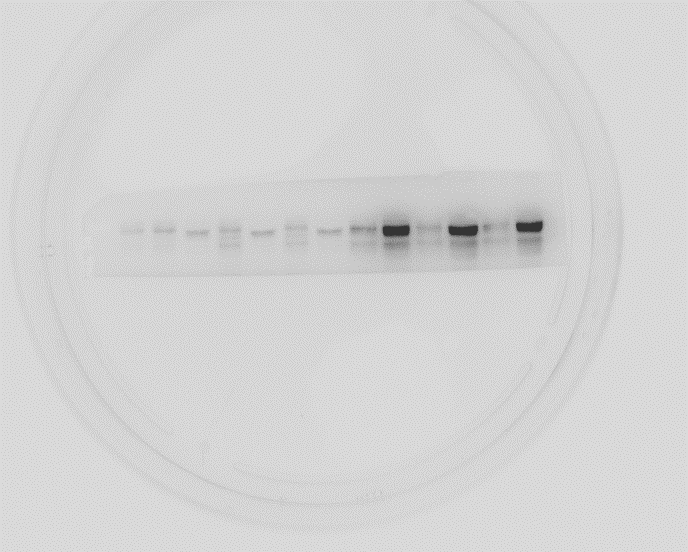

Supplement: DATA SHEET S1 — All western blots without being cropped in Figures 1–4, n >= 3. [file Data_Sheet_1.ZIP › full images of western blots/figure 3/TFEB.tif]

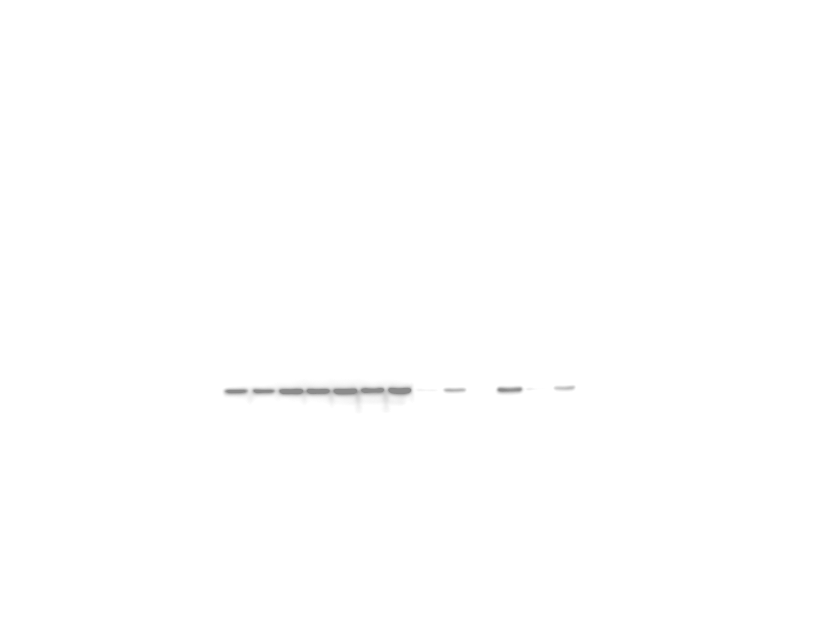

Supplement: DATA SHEET S1 — All western blots without being cropped in Figures 1–4, n >= 3. [file Data_Sheet_1.ZIP › full images of western blots/figure 3/tubulin.tif]

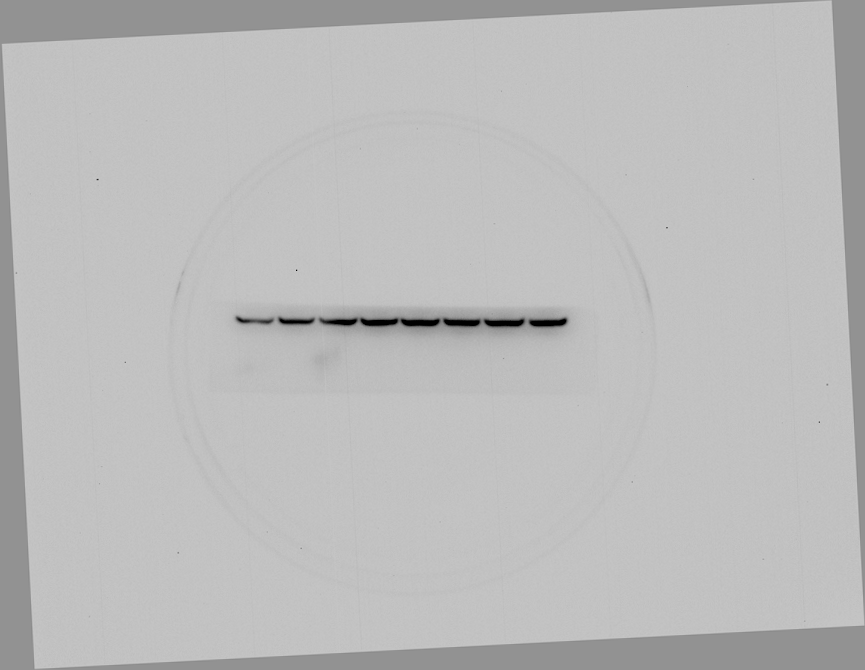

Supplement: DATA SHEET S1 — All western blots without being cropped in Figures 1–4, n >= 3. [file Data_Sheet_1.ZIP › full images of western blots/figure 4/A/actin.tif]

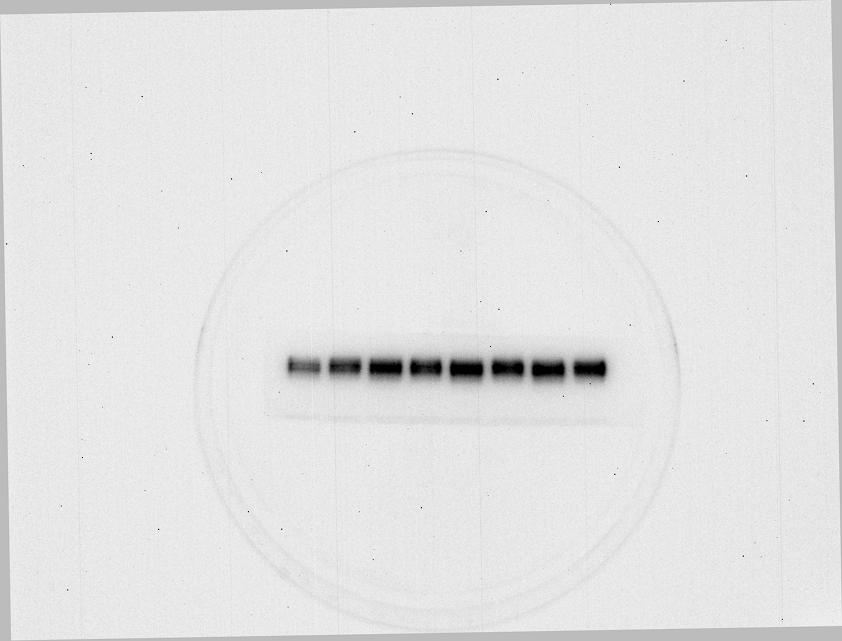

Supplement: DATA SHEET S1 — All western blots without being cropped in Figures 1–4, n >= 3. [file Data_Sheet_1.ZIP › full images of western blots/figure 4/A/LAMP-1.tif]

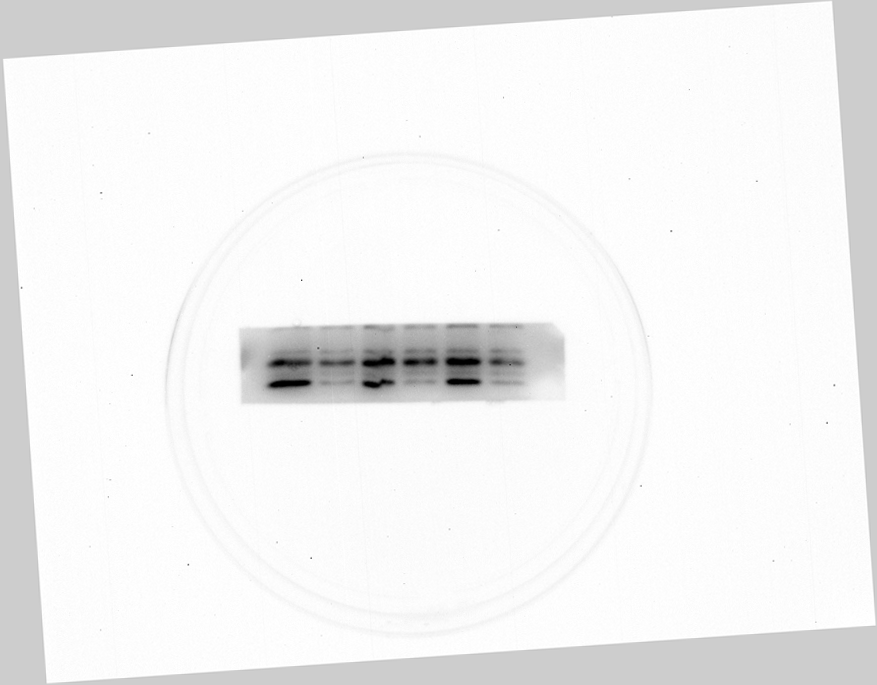

Supplement: DATA SHEET S1 — All western blots without being cropped in Figures 1–4, n >= 3. [file Data_Sheet_1.ZIP › full images of western blots/figure 4/A/LC3.tif]

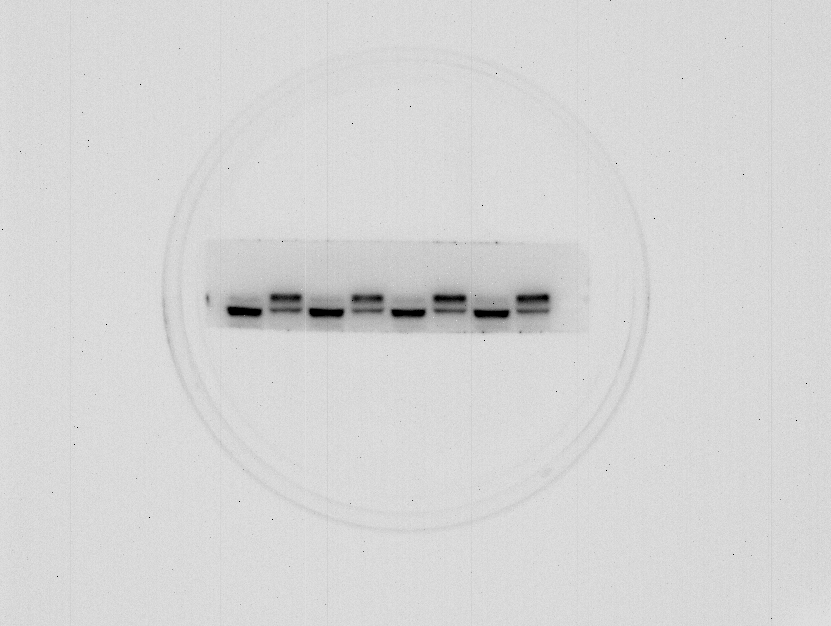

Supplement: DATA SHEET S1 — All western blots without being cropped in Figures 1–4, n >= 3. [file Data_Sheet_1.ZIP › full images of western blots/figure 4/A/TFEB.tif]

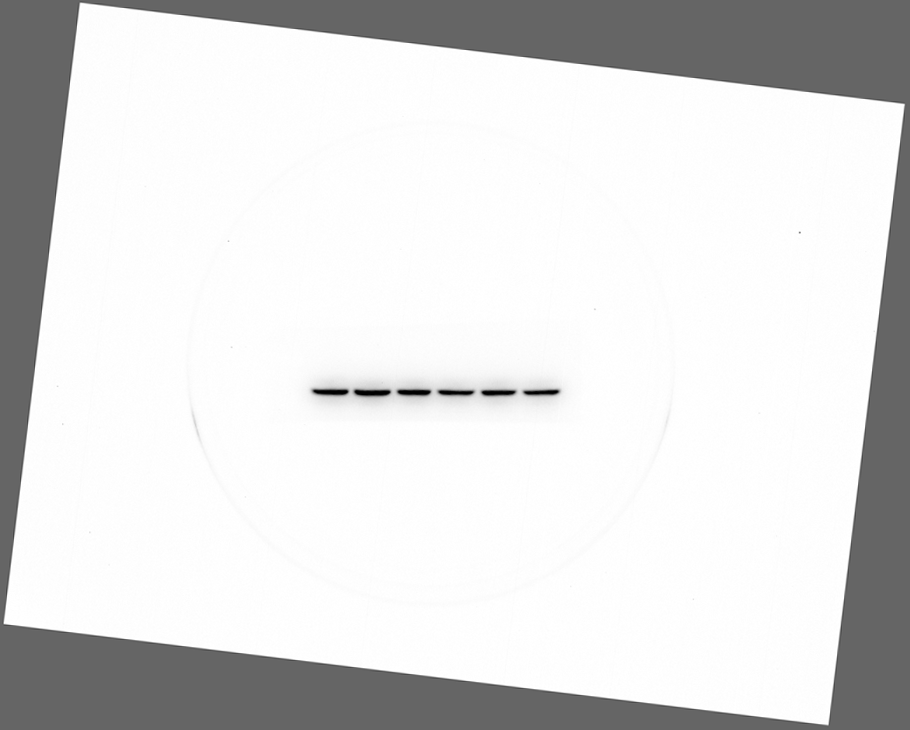

Supplement: DATA SHEET S1 — All western blots without being cropped in Figures 1–4, n >= 3. [file Data_Sheet_1.ZIP › full images of western blots/figure 4/H/4H-1/actin.tif]

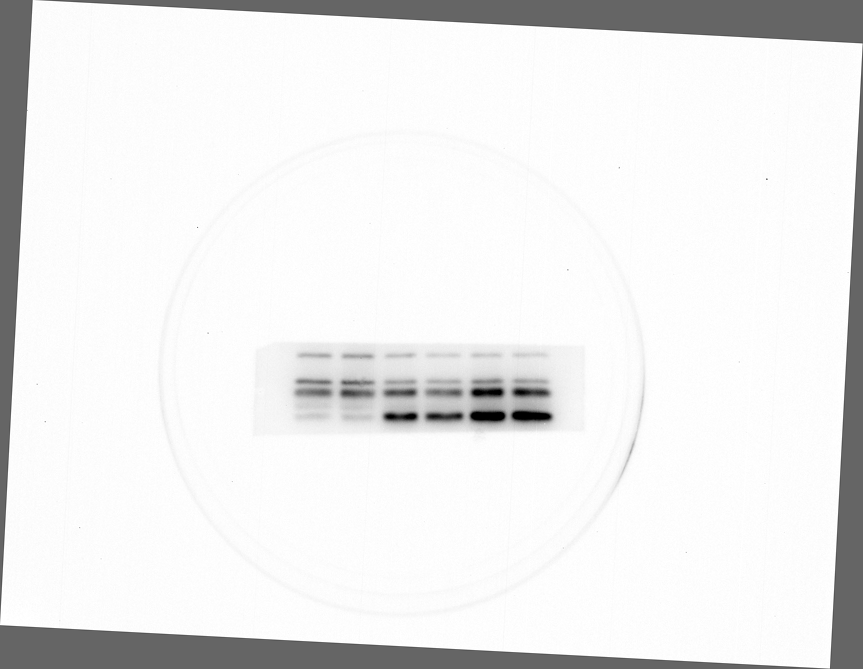

Supplement: DATA SHEET S1 — All western blots without being cropped in Figures 1–4, n >= 3. [file Data_Sheet_1.ZIP › full images of western blots/figure 4/H/4H-1/LC3.tif]

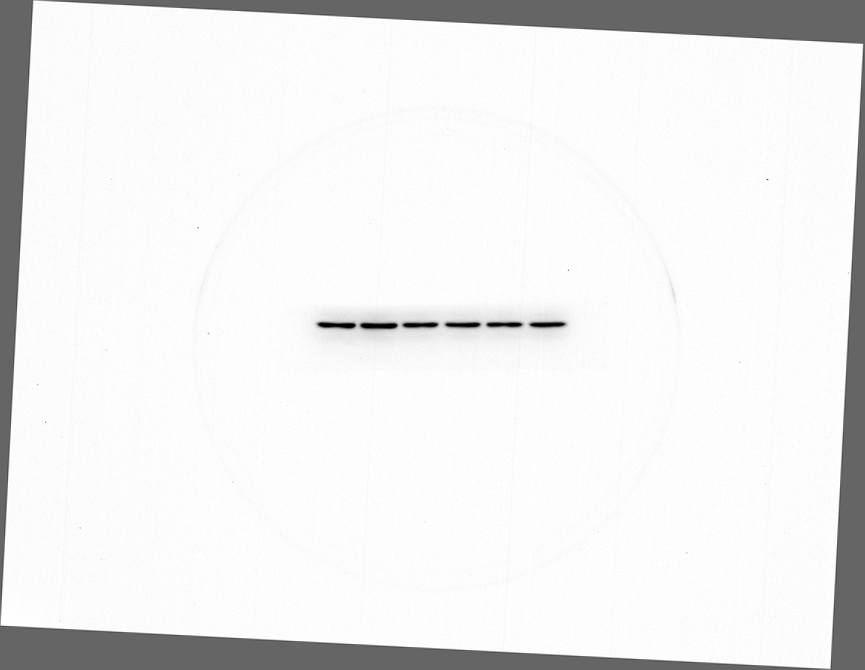

Supplement: DATA SHEET S1 — All western blots without being cropped in Figures 1–4, n >= 3. [file Data_Sheet_1.ZIP › full images of western blots/figure 4/H/4H-2/actin.png]

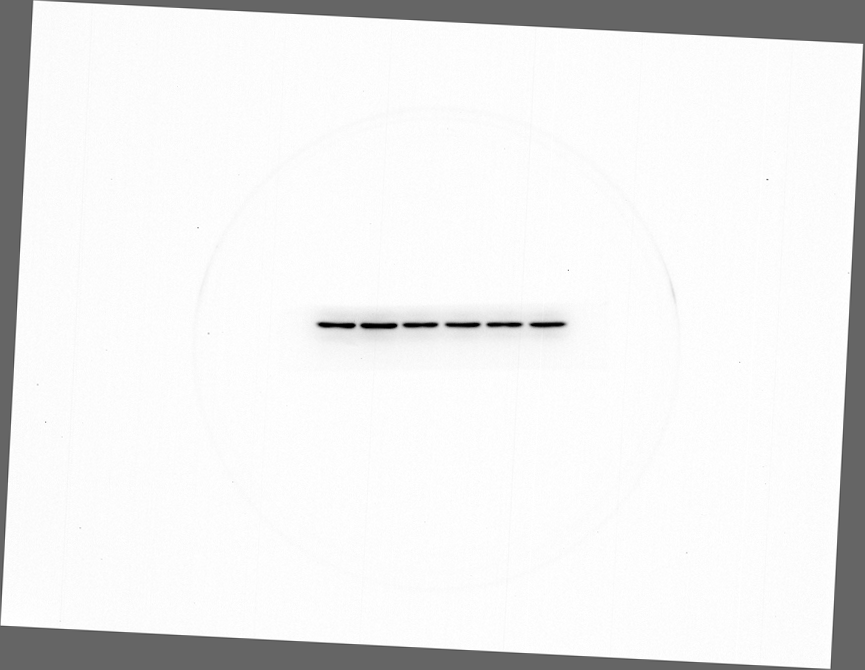

Supplement: DATA SHEET S1 — All western blots without being cropped in Figures 1–4, n >= 3. [file Data_Sheet_1.ZIP › full images of western blots/figure 4/H/4H-2/actin.tif]

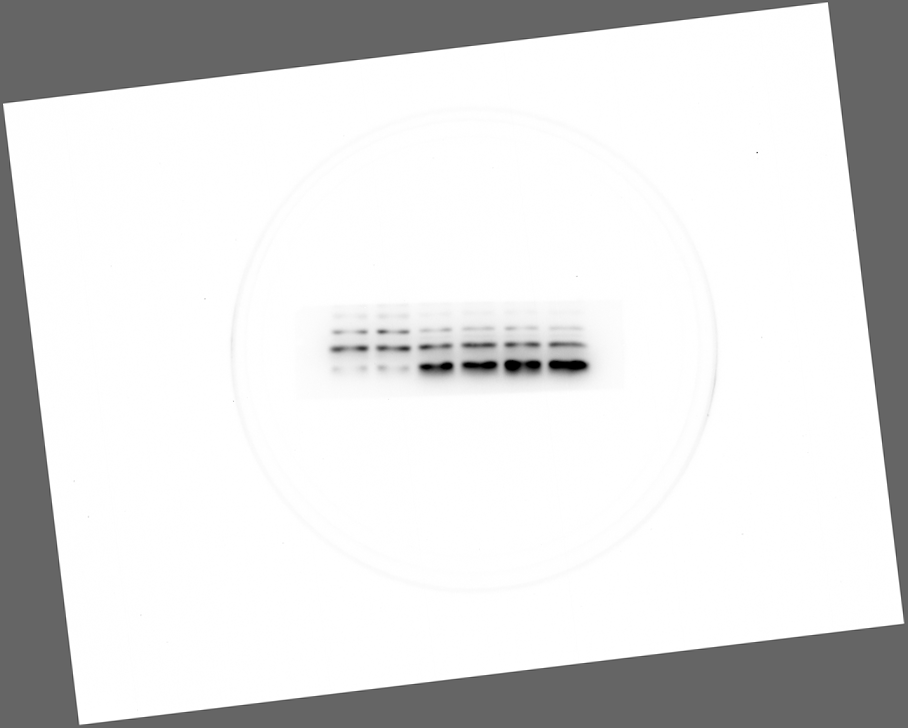

Supplement: DATA SHEET S1 — All western blots without being cropped in Figures 1–4, n >= 3. [file Data_Sheet_1.ZIP › full images of western blots/figure 4/H/4H-2/LC3.png]

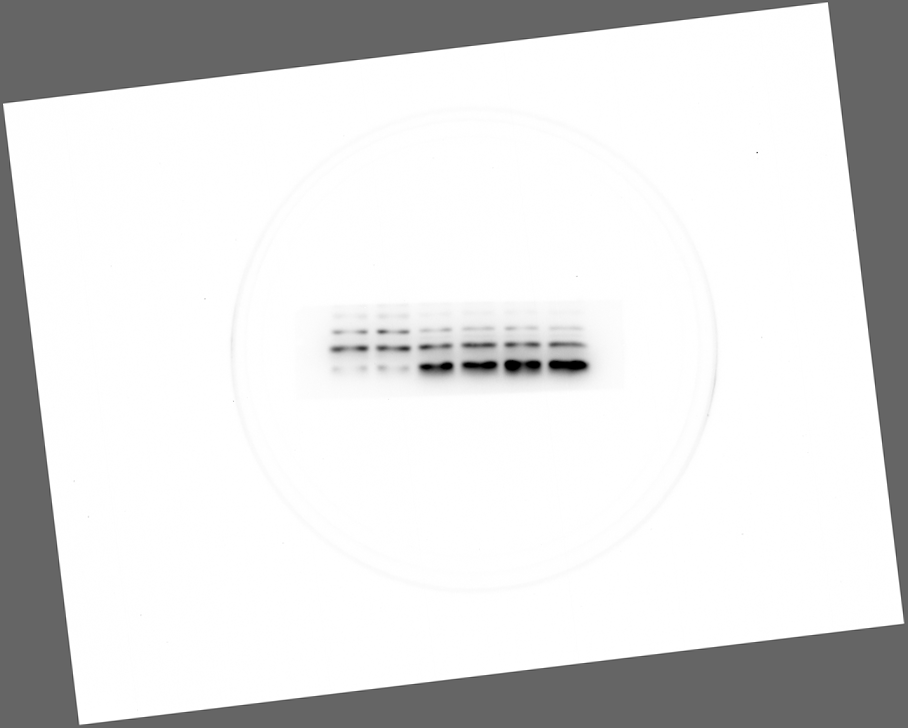

Supplement: DATA SHEET S1 — All western blots without being cropped in Figures 1–4, n >= 3. [file Data_Sheet_1.ZIP › full images of western blots/figure 4/H/4H-2/LC3.tif]
